# Effects of low-volume court-based sprint interval training on aerobic capacity and sport-specific endurance performance in competitive tennis players

**DOI:** 10.5114/biolsport.2025.139088

**Published:** 2024-08-08

**Authors:** Wenpu Yang, Langlang Yin, Eric Tsz-Chun Poon, Indy Man Kit Ho, Haochong Liu, Bing Qi, Qian Li, Yanchun Li

**Affiliations:** 1 Sports Coaching College, Beijing Sport University, Beijing; 2 School of Physcial Education, Bohai University, Jinzhou, China; 3 China Institute of Sports and Health Science, Beijing Sport University, Beijing, China; 4 The Education University of Hong Kong, No. 10 Lo Ping Road, Tai Po, New Territories, Hong Kong SAR, China; 5 Hong Kong Metropolitan University, Hong Kong, China; 6 Asian Academy for Sports and Fitness Professionals, Hong Kong, China

**Keywords:** Elimination rate of blood lactate, Tennis training, YoYo-IR2, Repeated sprint ability, Maximum oxygen uptake, Sports specific training

## Abstract

Sprint interval training (SIT) is a potent exercise strategy to enhance athletes’ aerobic capacity in a time-efficient manner. The purpose of this study was to examine the effect of a low-volume court-based SIT program on aerobic capacity and sport-specific endurance performance for competitive tennis players. Sixteen competitive collegiate tennis players were randomly assigned to the SIT (court-based repeated-sprint training) and traditional endurance training (ET; 45-min continuous treadmill running) groups for a 6-week intervention (3 sessions/week). The maximal oxygen uptake ($\dot{\text{V}}$O_2max_), minute ventilation at peak exercise (VEmax), ventilatory anaerobic threshold in percentage of $\dot{\text{V}}$O_2max_ (VT/VO_2_), and elimination rate of blood lactate (BLA_er_) were assessed, whereas the Yo-Yo Intermittent Recovery Test Level 2 (YoYo-IR2) and the tennis-specific HIT&TURN test were conducted at baseline and after the intervention. Both SIT and ET showed significant improvements in $\dot{\text{V}}$O_2max_ (p < 0.01) with moderate effect sizes (ES = 0.64 and 0.98, respectively), as well as in VE_max_ (p < 0.01) with small effect sizes (ES = 0.23 and 0.21, respectively), and VT/VO_2_ (p < 0.01) with large effect sizes (ES = 2.37 and 3.85, respectively). The BLA_er_ improved significantly in SIT (ES = 1.03; p < 0.05) whereas no significant changes occurred in ET. The magnitude-based decision showed a clear and superior improvement in both YoYo-IR2 (ES = 0.69) and HIT&TURN (ES = 1.72) tests in SIT than ET. Compared with traditional ET, court-based SIT can be a time-efficient strategy to improve aerobic capacity and tennis-specific endurance without requiring specialized equipment for competitive tennis players.

## INTRODUCTION

Tennis is a sport requiring both explosive, high-intensity efforts (e.g., acceleration or reacceleration for sprinting, and deceleration for stopping and turning) and sustained bouts of lower-intensity activity (e.g., rallies and endurance-based movements) [1, 2]. To cope with the high level of physical, technical, and tactical demands of performance, competitive tennis players need a combination of fitness qualities, such as speed, agility, repeated-sprint ability (RSA), and power, combined with well-developed aerobic fitness [3]. Typical tennis competitions have average points lasting less than 10 s, with rest periods of approximately 20 s between points and 90 s after every second game whereas, the total match time can be lasted for hours (occasionally more than 5 hours) [1] and therefore, it can also be highly challenging to the metabolic systems for competitive players.

Despite the high demand for explosive movements in a tennis serve, ground stroke, and repeated sprints, it has been shown that tennis is aerobic-dominant with about 70% aerobic and 30% anaerobic energy supply during the game [4]. In addition, high-intensity tennis drills heavily rely on both the phosphagen and glycolytic energy systems for rapid fuel supply [5]. As oxidative phosphorylation is one of the key processes for the resynthesis of phosphocreatine, athletes with good aerobic capacity can demonstrate better phosphagen reserve [6], and are more efficient in removing metabolic by-products for facilitating recovery and the maintenance of high performance in subsequent sets and games [7].

Traditionally, continuous endurance training (ET) such as moderate to high-intensity long-distance running (e.g. at 75–85% of lactate threshold velocity or 70% of maximal oxygen uptake [$\dot{\text{V}}$O_2max_]) was shown to be highly effective in inducing numerous training adaptations (e.g., increasing $\dot{\text{V}}$O_2max_) to improve aerobic exercise capacity and sports performance [8, 9]. Nevertheless, studies showed that moderate-intensity, continuous-based ET did not provide comparable improvement in the anaerobic capacity as the high-intensity intermittent training (HIIT) [9, 10]. Moreover, the traditional ET may not be effective and efficient enough to achieve adaptations to meet the complex physical demands of sports with limited training time. Meanwhile, it may lack the specificity to maintain training qualities and stimuli of speed and power in many sports [11–13]. In this regard, sprint interval training (SIT) (an intense variant of HIIT) has emerged as an effective and time-efficient training method that can enhance physical performance in competitive sports especially those highly demanding on repeated and intermittent sprints.

SIT involves short bursts of exercise with “all out” effort (i.e., at a power output or velocity above $\dot{\text{V}}$O_2max_) interspersed with periods of rest or low-intensity activities [14]. It can improve both the aerobic and anaerobic performance of athletes in intermittent sports with substantially lower overall training volume [1]. Due to the similar nature of intermittent sprinting activities in many racquet or team sports, SIT is likely to maximize the training transfer and the benefit of performance enhancement in the game condition. Moreover, SIT often evokes similar or even superior physiological adaptations and exercise improvements compared to traditional ET [15, 16], making it one of the most effective forms of exercise for improving physical performance in various intermittent team sports [13], including soccer, basketball, volleyball, and field hockey [17]. Given the similar frequent starts and stops nature in tennis, improving the ability to repeatedly perform high-intensity efforts and to recover rapidly of competitive tennis players are the keys to success [18]. For these reasons, physical training should be optimized to enhance both aerobic and anaerobic performance as part of any tennis conditioning program.

In spite of the aforementioned potential benefits, the application of SIT in intermittent sports like tennis, particularly among elite athletes remains relatively unexplored. Existing research on SIT has primarily investigated endurance sports, such as cycling and running performance in triathlon [19], and has recruited general populations rather than elite athletes. Prior systematic reviews and meta-analyses have made efforts to examine the impact of cycle ergometer-based SIT on aerobic capacity and sprint power [14]. However, these synthesis studies mostly incorporated a significantly limited amount of data (less than 10%) from competitive athletes. Moreover, the populations involved in these studies were predominantly composed of sedentary individuals or those engaged in only moderate-frequency recreational activities [14], suggesting a limited application of findings to the elite populations. In addition, previous research has primarily relied on laboratory-based equipment such as cycle ergometers and treadmills [20]. However, these modes of training and testing may not fully replicate the specific demands and conditions of tennis. Moreover, using such equipment can be both expensive and time-consuming, particularly when multiple athletes are involved in the training at the same time [21]. Given the constraints of time and resources that are often associated with high-performance training, these interventions may be deemed impractical for some athletes and coaches. Furthermore, if training activities replicate the motor patterns, contraction types, and force patterns utilized during competitive performance, there is a higher likelihood of achieving enhanced training transfer to on-field performance [22]. In contrast to the lack of specificity in ergometer-based protocols, court-based interventions should theoretically better develop the physical, physiological, and metabolic indices required for field-based sports including tennis [23].

To fill the research gaps, this study aimed to explore the effect of a 6-week court-based SIT on improving aerobic capacity and repeated sprinting ability for competitive tennis players, utilizing both laboratory and tennis-specific field tests. It was hypothesized that SIT would induce a greater improvement in these parameters compared with traditional continuous-based ET. For digging into the principle of the application of SIT boosting the transformation from training to on-field performance, the mechanism of oxygen uptake and transport should also be taken into account.

## MATERIALS AND METHODS

### Subjects

Sixteen competitive collegiate tennis players volunteered to participate in the study during the preparatory season. Based on the requirement for maintaining consistency among the characteristics of the participants and the shortage of female athletes from the tennis academy, the study chose to focus exclusively on male tennis players. The sample size of the study was determined to use a priori power analysis (utilizing G*Power version 3.1.9.7, University of Dusseldorf). The analysis considered several parameters, including the effect size (ES) index (0.40) assuming a large partial eta-squared (.14), α error probability (.05), power (0.90), number of groups (2) and measurements (3), and correlation among repeated measurements (.5). Considering these parameters, the power analysis indicated that a sample size of 16 subjects was appropriate for the study [13].

The participants, who were all right-handed, had a weekly training volume of 20 hours consisting of 3 hours of technical and tactical tennis practice and 1 hour of physical conditioning on weekdays. The inclusion criteria for participants are as follows: all subjects were in good health and had no severe injuries during the last six months before the study and had a minimum of 4 years of systematic tennis training experience. Participants were fully informed of the experimental procedures, benefits, and risks associated with the study before giving their written informed consent to participate. The tests were conducted at least 48 hours after a competitive match or heavy training session. The subjects participated in all the training sessions as well as pre-and post-tests. The study was approved by the Research Ethics Committee of Beijing Sport University (Approval number: 2023210H) and all procedures were conducted following the Declaration of Helsinki.

### Design

A longitudinal and randomized controlled experimental design was used to investigate the effect of a 6-week court-based SIT intervention on the aerobic ability and performance parameters of tennis players in 2023. A 2-group, repeated measures (pre-test and post-test) design was used. Subjects were randomly allocated into the SIT and endurance training (ET) groups (SIT: n = 8, ET: n = 8) using stratified block randomization. The demographic data of the subjects are presented in Table 1. No significant differences were observed among the groups in terms of competition level, biometric training characteristics, aerobic parameters, and aerobic-specific performance before intervention. Besides the intervention, both groups maintained the same amount of regular physical and technical-tactical training agreed upon by the tennis academy. No missing sessions or injuries were reported during the intervention period.

**TABLE 1:** Physical characteristics of tennis players included in the analysis (baseline).

	SIT (n = 8)	ET (n = 8)	*p*
Age (year)	22.24 (3.44)	22.00 (1.71)	.93
Height (cm)	179.63 (6.89)	174.88 (4.16)	.12
Body mass (kg)	74.73 (8.79)	69.08 (5.99)	.16
Training background (year)	6.00 (1.48)	5.38 (1.51)	.47
Competitive level (ITN)	4.14 (1.01)	4.26 (0.97)	.81

Abbreviations: ET, endurance training as the control group; SIT, sprint interval training group; ITN, international tennis number. Note: Values are presented as mean (SD) and p-value of the differences between SIT and ET.

### Methodology

**FIG. 1:**
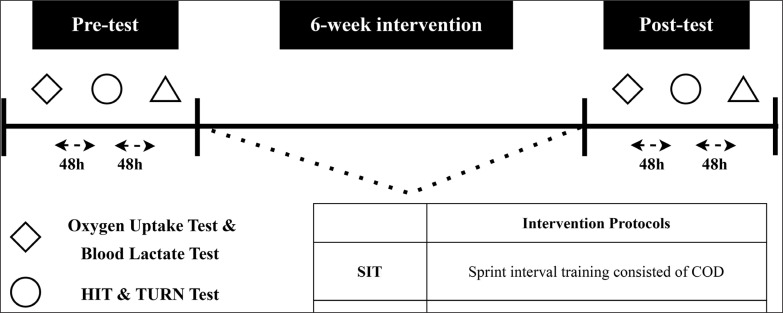
Schematic diagram of the experimental process.

The experimental flow is shown in Figure 1. Prior to the start of the intervention, all participants were required to perform the $\dot{\text{V}}$O_2max_ and blood lactate tests as well as two tennis field-based endurance tests including Hit and Turn Test (HIT&TURN), and YO-YO Intermittent Recovery Test Level 2 (YoYo-IR2). All the field-based tests and court-based SIT were performed at the outdoor hard surface tennis court. For each training and testing session, the subjects followed a 10-minute standardized general warm-up and 10-minute cool-down protocol including jogging, skipping, dynamic warm-up, and stretching. Participants refrained from intensive exercise for a minimum of 48 hours before the protocol. Additionally, no strenuous physical activity was undertaken within 48 hours preceding the testing sessions. The research took place during periods of preparatory training period that did not involve competition, and all measurements were conducted in the morning, typically between 7:30 a.m. and 8:30 a.m.

#### Laboratory Measurements

An incremental treadmill protocol (H/P Cosmos, Germany) was used to determine the related aerobic parameters, including $\dot{\text{V}}$O_2max_, minute ventilation at peak exercise (VEmax), and ventilatory anaerobic threshold in the percentage of $\dot{\text{V}}$O_2max_ (VT/VO_2_), using an online breath-by-breath gas collection system (Max I, America). The test began with an initial velocity of 6 km/h, increasing by 1 km/h every minute until subjective exhaustion, with a constant gradient of 1.5%. Expired air was continuously analyzed for gas volume, O_2_ concentration, and CO_2_ concentration. Before each test day, the system underwent volume calibration to ensure accurate measurements, and gas calibration was performed before each test followed the manufacturer’s provided instructions. A blood lactate elimination test was also conducted to analyze the recovery speed of athletes after increasing load and to evaluate their recovery ability after aerobic exercise. Blood samples were collected for rest (before testing with players being seated) and 0, 1, 3, 5, 7, 10, and 15 min immediately after the increasing load test via a volume of 20 microliters of fingertip blood. The automatic blood lactate analyzer (EKF-diagnostic GmbH, Barleben, Germany) was used to measure blood lactate, whereas the elimination rate of blood lactate (BLA_er_) was calculated by the following formula [24]: BLA_er_ = (BLA_max_-BLA_15_)/(15-T), where BLA_max_ is the peak blood lactate value, BLA_15_ is the blood lactate value of 15 min, and T is the time of peak blood lactate production. The heart rate (HR) was monitored and recorded at 5-second intervals during the exercise (S610, Polar Electro, Kempele, Finland), and maximal HR (HRmax) was determined as the highest 5-second mean value. The rating of perceived exertion (RPE) was obtained using the Borg RPE Scale (scale 6–20) [25].

#### Hit and Turn Test

**FIG. 2:**
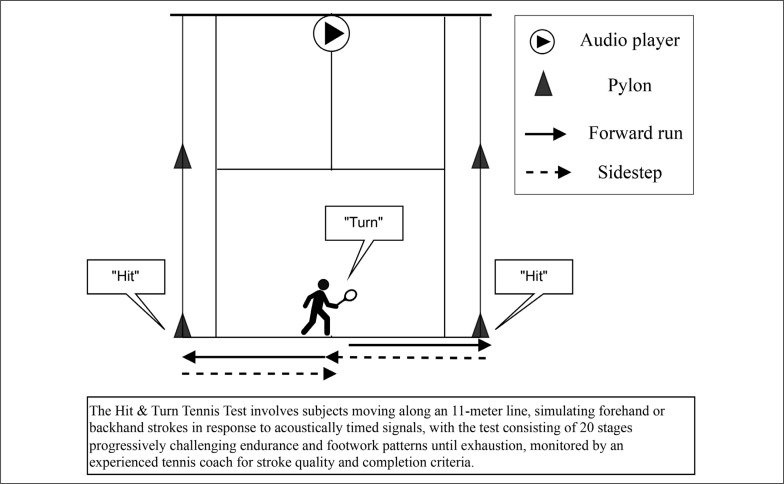
Running and Hitting Court Position during the Hit and Turn Test.

HIT&TURN is a valid and reliable progressive on-court fitness test to assess tennis-specific endurance. It is designed to be performed individually or simultaneously by multiple players incorporating acoustic control. (Figure 2) [26]. The test involves specific movements along the baseline, such as sidesteps and running, combined with simulated forehand and backhand strokes at the doubles court corner, which is located at a distance of 11.0 meters. At the start of each test level, players positioned themselves in the middle of the baseline, facing the net, with their racket in front of the body. Upon hearing a signal, players turned sideways and sprinted to the designated backhand or forehand corner as indicated by a CD player. After executing their shot, they returned to the middle of the court using side steps or crossover steps while maintaining their focus on the net. As they passed the middle of the baseline, they turned sideways once again and continued running towards the corner on the opposite side of the court from their starting position. The test was terminated when players were unable to reach the pylons within the allocated time or were no longer able to perform the required movement patterns. The highest completed level was used to determine the tennis-specific endurance capacity [26]. The RPE is recorded both at the beginning and at the end of the test to gauge the subjective perception of effort.

#### YO-YO Intermittent Recovery Test Level 2

YoYo-IR2 test was used as a reliable method to verify the ability of trained athletes to perform repeated bouts of high-intensity interval runs with high anaerobic energy contribution [27]. The YoYo-IR2 test provides a simple and valid way to obtain important information about an individual’s capacity to recover from repeated intense exercise and to examine training effects on performance. After the completion of the standard dynamic warm-up, participants sprinted 2 × 20 m at progressively increasing speeds and jogged around a marker placed 5 m behind the finish line for 10 seconds during every 40 m-shuttle (controlled by audio signals). The test ended when the participant chose to terminate it, or when the subject was unable to complete the shuttle run in time on two consecutive occasions. The final running distance was recorded for analysis.

#### Interventions

**FIG. 3:**
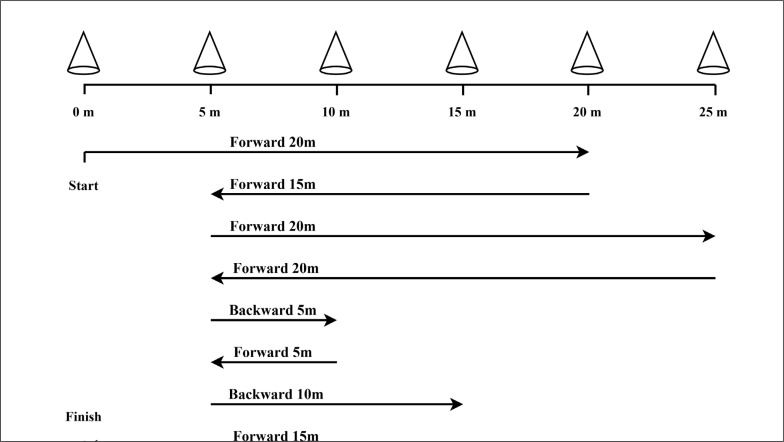
Sprint interval training training protocol.

Participants commenced the training protocol 48 hours following the final pre-test visit. The training involved three sessions of traditional continuous-based ET or court-based SIT per week on alternate days (i.e., Monday, Wednesday, and Friday) for six weeks. Our ET protocol was modified from a previous study and agreed upon by the coaching and medical team such that the intensity and volume of the selected ET protocol were sufficient to promote the aerobic fitness of participants without the risk of overtraining [13]. It consisted of 45 min of continuous treadmill running at a velocity corresponding to 75% $\dot{\text{V}}$O_2max_. Before and after each ET session, a 10 µL blood sample was taken from the fingertip to determine the blood lactate concentration. The SIT protocol (approximately a total of 20 to 21 minutes including the recovery period) modified from a previous study involved three sets of high-intensity sprints interspersed with short recovery periods [13]. Each interval run was 110 m in total distance, and involved forward and backward sprints over distances ranging from 5 to 20 m with multiple changes of direction (COD) (Figure 3). A set consisted of 3 × (110 m sprint with a 20 s recovery period between each sprint) and a 5 min recovery period between sets. Before the commencement of the SIT protocol and at the end of each run, a 10 µL blood sample was taken from the fingertip to determine the whole blood lactate concentration. Participants were verbally encouraged through-out both exercise protocols. All training sessions for both groups were supervised by an investigator with strength and conditioning experience. The Polar Team2 System (Polar Electro Oy, Kemple, Finland) was used to monitor the heart rate of each player throughout each training session, with data later extracted from custom-specific software (Polar Team2, Electro Oy, Kemple, Finland), to obtain HRmax, time spent in each HRmax% zone and training impulse (TRIMP). TRIMP takes into account the training duration and intensity at the same time and reflects the comprehensive effect of training on the internal and external load of the athlete’s body, as well as the load of medium and high-intensity training. The method to determine the athlete’s TRIMP in the current study is based on the formula proposed by Edwards, which means a weight factor of each heart rate zone is given whereas the TRIMP per each zone is acquired by multiplying the exercise time. The HR_max_ of each player was established using the peak value recorded by the monitoring system during the training.

### Statistical Analysis

Experimental data were processed by the SPSS statistical software package (version 25.0, Chicago, IL, USA); all test results were reported as mean (standard deviation). The normality and homogeneity of variances of the tests (Shapiro-Wilk test) results were checked before the subsequent analyses. Independent t-tests in biometrics, training characteristics, aerobic and lactate parameters, and HIT&TURN level were used to assess differences before training intervention (week 0) among the 2 groups. A 2-way repeated measures analysis of variance (ANOVA) was then used to compare the within (time, pre-test vs post-test) and between-group (SIT vs ET) difference to determine the effects of interventions on $\dot{\text{V}}$O_2max_, BLA_er_, HIT&TURN level, and YoYo-IR2. Whenever a significant difference was detected for either of the main effects, a post hoc analysis using the least significant difference (LSD) method was conducted. The partial eta-squared (*η*^2^) effect size measure was computed to assess the magnitude of the main and interaction effects in the analysis of variance. The partial *η*^2^ values of .01 to .05, .06 to .13, and > .14 represented a small, medium, and large effect size, respectively. Besides, the effect size (ES) was calculated using Cohen’s d to quantify the magnitude of pre- and post-intervention change and to reflect the comparison of training effects within SIT and ET groups based on the following scales: < 0.2 trivial, 0.2–0.6 small, 0.6–1.2 moderate, 1.2–2.0 large and > 2.0 very large [28].

Meanwhile, the non-clinical magnitude-based decision (MBD) was also performed to provide additional insights into between-group comparisons. The MBD was performed by Rstudio software (with the library “mbir”) using the precision of estimation to compare the mean changes (post-test – pre-test) between ET and SIT groups, via respective 95% confidence intervals (CI) and the standardized effect. The effects were deemed unclear if the corresponding 95% CI crossed the thresholds of the effect regarded as substantially positive and negative by > 5% while the qualitative inference and probabilistic classifications followed previously established guidelines [28].

## RESULTS

### Laboratory Measurements

**FIG. 4:**
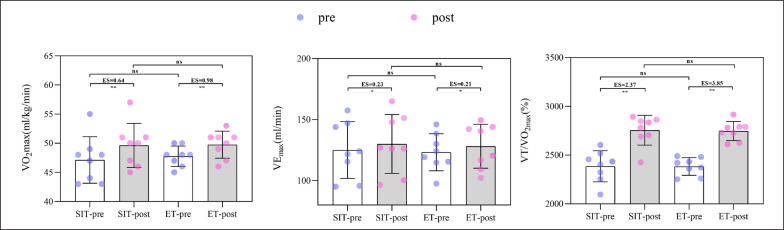
Effects of sprint interval training and endurance training on laboratory measurements.

The physiological variables analyzed ($\dot{\text{V}}$O_2max_, VEmax, VT/VO_2_) at baseline and after 6 weeks of intervention were shown in Figure 4. There were significant main time effects for $\dot{\text{V}}$O_2max_, VEmax, and VT/VO_2_, with a significant and moderate increase in the $\dot{\text{V}}$O_2max_ of SIT (*p* < 0.01, ES = 0.64) and ET (*p* < 0.01, ES = 0.98), a significant and small increase in the VE_max_ of SIT (*p* < 0.01, ES = 0.23) and ET (*p* < 0.01, ES = 0.21), a significant and very large increase in the VT/VO_2_ of SIT (*p* < 0.01, ES = 2.37) and ET (*p* < 0.01, ES = 3.85) for both SIT and ET, respectively. Moreover, there was no significant group × time interaction, for all Laboratory parameters in all the groups analyzed (SIT or ET) (*p*< 0.05). Similarly, MBD showed no between-group significant difference or clear effect.

### Court-based Aerobic performance

A significant group × time interaction (*p* = 0.01) was found in the HIT&TURN performance (Table 2) while the main effect for time was also significant (*p*<0.001). There was a significant increase in the maximum level achieved in the HIT&TURN for SIT, (22.7% increase, *p* = 0.014, ES = 1.83, large) between pre- and post-test whereas, ET showed some but not statistically significant increase (6.6% increase, *p* > 0.05, ES = 0.67, moderate). There was a significant main effect for time in YoYo-IR2 performance (8.3% increase, *p* = 0.02, ES = 0.70, moderate) and BLA_er_ (13.1% increase, *p* = 0.02, ES = 0.70, moderate) in SIT but no significant change in ET was observed. No group × time interaction was found for YoYo-IR2 distance (*p* = 0.17) and BLA_er_ (*p* = 0.50). Regarding the RPE in the HIT&TURN test, it ranged from 18.0 to 18.9 whereas no significant change in both within the group (pre- and post-test) and between-group comparisons (*p* > 0.05).

**TABLE 2:** Effects of sprint interval training and endurance training on performance variables analyzed.

Variable	Group	Pre-test	Post-test	Time		Interaction	

				*p*	*η* ^2partial^	*p*	*η* ^2partial^	ES (d)
BLA_er_(%)	SIT	25.50 ± 13.85	38.63 ± 11.64^*^	0.02	0.35	0.50	0.03	1.03 moderate
	ET	29.38 ± 9.81	37.94 ± 9.84					0.87 moderate

YoYo-IR2 distance (m)	SIT	725.00 ± 96.65	785.25 ± 73.87^*^	0.02	0.53	0.17	0.13	0.70 moderate
	ET	727.50 ± 161.40	755.00 ± 135.96					0.18 trivial

HIT&TURN (level_max_)	SIT	13.38 ± 1.30	16.38 ± 1.92^*^	0.00	0.76	0.01	0.48	1.83 large
	ET	13.75 ± 1.28	14.62 ± 1.30					0.67 moderate

Abbreviations: ET, endurance training; SIT, sprint interval training group; ES, effect size; BLAer, elimination rate of blood lactic acid.

Note

* : significant difference observed with p < 0.05.

**FIG. 5:**
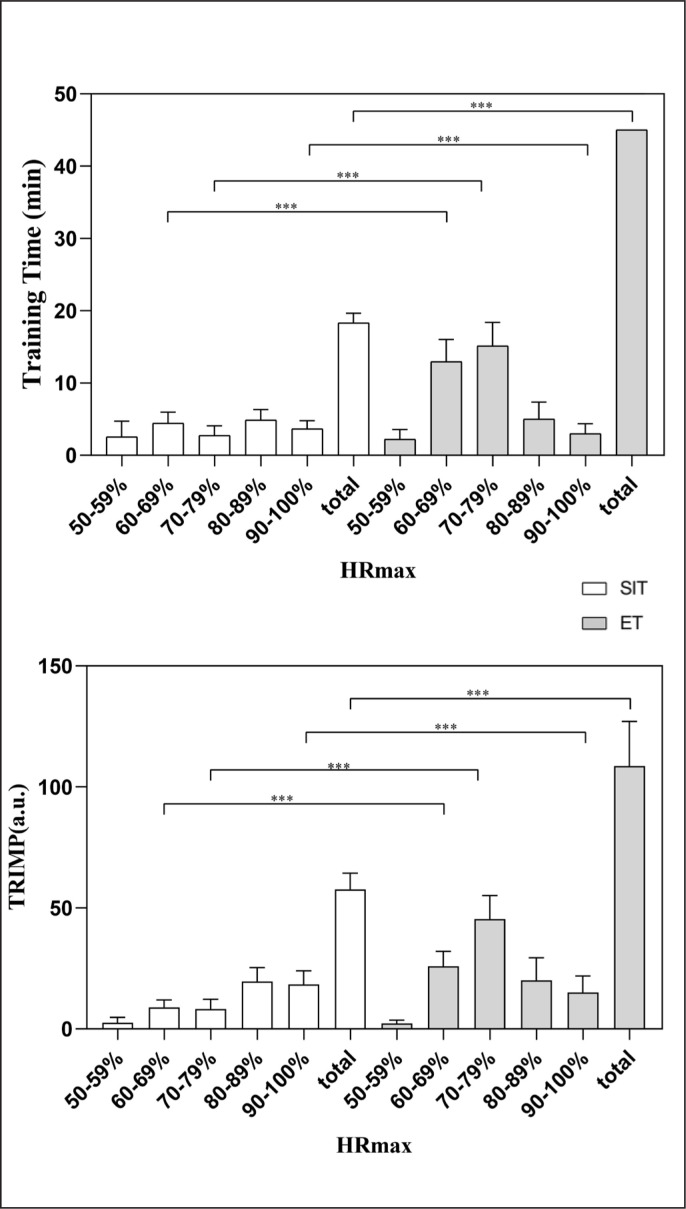
Percentage of time spent and TRIMP by players in the different heart rate (HR) categories during SIT and ET training sessions.

MBD showed an “almost certainly large” increase (ES = 1.72) in the HIT&TURN improvement in SIT. For the YoYo-IR2, the MBD concluded a “likely moderate” increase in mean change (ES = 0.69) in SIT compared to the ET group. The mean TRIMP of the players obtained in this study was 58.87 ± 6.93 a.u. and 108.39 ± 17.03 a.u. for the SIT and ET, respectively. Figure 5 shows the percentage of time spent by the players in the different HR categories and corresponding TRIMP during the training sessions performed in the study. During the 6-week training, the mean weekly effective training time (time spent within 50–100% HR_max_ zone) and TRIMP in the 80–100% HR_max_ intensity range of the SIT group were significantly higher than those in the ET group (*p* < 0.05), while the total weekly effective training time and TRIMP for the SIT were significantly lower than the ET (*p* < 0.05).

## DISCUSSION

To the best of our knowledge, this study is the first to investigate the effects of court-based SIT on both laboratory and field-based aerobic capacity outcomes for competitive tennis players. Our major finding was that with a court-based, bidirectional sprinting protocol, the lower-volume SIT, compared with the conventional equipment-based ET, played an important role in laboratory-based aerobic capacity and potentially more superior tennis-specific field-based endurance performance. In particular, SIT made practical improvements in conventional cardiorespiratory parameters ($\dot{\text{V}}$O_2max_, VEmax, and VT/VO_2_), and lactate elimination like ET. Moreover, it showed superior improvements in tennis-specific endurance (HIT&TURN and YoYo-IR2). It was significant that, these benefits were obtained in a much shorter time frame (i.e., ~90% less total exercise time or only 1/3 of the ET in terms of total training duration) than traditional ET and without requiring specialized equipment, making SIT a practical and efficient conditioning method act as a best suited for the tight training schedule of competitive tennis players.

A high level of cardiorespiratory function is essential for prolonged high-intensity movements in competitive tennis, as it allows for better oxygen delivery to working muscles [2]. Our findings of increased $\dot{\text{V}}$O_2max_ following both SIT (7.2%) and ET (5.4%) are consistent with those from previous meta-analyzes [29, 30]. Although the central and peripheral physiological adaptations associated with increased $\dot{\text{V}}$O_2max_ have been widely recognized for a long time [31], more recent studies have shed light on the distinct mechanisms through which SIT and ET induce adaptive changes in the body. In particular, it has been suggested that SIT primarily improves peripheral mechanisms, which is mainly due to the increase in skeletal muscle arterial-venous oxygen difference (a-vO2 diff), related oxidative enzymes in the muscles, and the density and volume increases in mitochondria [12]. Gibala and McGee [32] supported this notion by suggesting that improvements in endurance performance reported after only two weeks of SIT serve as possible evidence that peripheral adaptations are primarily responsible, considering the absence of any noticeable improvement in $\dot{\text{V}}$O_2max_. Similarly, Macpherson and others [33] showed that 6 weeks of ET were more effective than SIT in increasing stroke volume and maximal cardiac output, whereas a group by time interaction in skeletal muscle a-vO2 diff favoring SIT was observed, suggesting that $\dot{\text{V}}$O_2max_ improvement following SIT was attributed to peripheral adaptations. These findings, in conjunction with ours, support a view that the use of SIT, an alternative training method, induces physiological adaptations for $\dot{\text{V}}$O_2max_ enhancement.

In addition to the improvements in maximal cardiorespiratory tests, SIT also showed significant improvements in blood lactate elimination capacity and YoYo-IR2 tests, as well as a clear enhancement in the HIT&TURN test. These tests are considered more sensitive measures of alterations in performing intermittent sports than $\dot{\text{V}}$O_2max_ [12, 27]. Our findings suggested that SIT can induce an improved tolerance to blood lactate accumulation, and increased abilities to repeatedly perform high-intensity exercise among competitive tennis players. With increasing exercise intensity during exercise, glycolytic metabolism also increases, and the lactate produced in muscles increases, subsequently entering the blood [34]. It has been shown that prolonged SIT programs increase the activity of enzymes of the glycolytic energy supply system, such as lactate dehydrogenase and glycogen phosphorylase, as well as enhance the buffering capacity of skeletal muscles [35]. These adaptive responses to the supra-maximal intensity of SIT are beneficial for competitive tennis players to adapt to the high-intensity confrontation during the game and accelerate their body recovery during the rest period of the game, thus enabling them to achieve better game performance. Based on the results of this study, SIT not only can develop the aerobic capacity of the body, but also the ability of the glycolytic system to supply energy. This can push forward the improvements in energy system efficiency and lactate elimination, which helps to delay the onset of fatigue during long and intense matches, giving tennis players more competitive advantages. Our findings are in line with a previous study using an 8-week ergometer-based and multi-ball SIT protocol on elite badminton players, which also revealed a better improvement in YoYo-IR2 and blood lactate elimination than traditional ET. Taken together, SIT can be considered as a low-volume alternative to substitute the traditional ET when the training schedule is tight, and assist ET in maximizing peripheral adaptations such as blood lactate elimination for better aerobic capacity in intermittent sports.

Another major finding in this study is that court-based SIT showed significantly greater improvements than ET (i.e., ES = 1.83 and ES = 0.67 for SIT and ET, respectively) and a likely moderate increase (i.e., ES = 0.69 in MBD for between-group comparison) in the tennis-specific endurance test (i.e. HIT&TURN), despite the similar improvements in $\dot{\text{V}}$O_2max_ values for both groups. The HIT&TURN test simulates the environment feedback, pace movement, ball striking, interval timing, and intensity of the actual tennis game, and therefore reflects the actual on-court performance of the tennis players during the game [26]. Our finding is consistent with the notion that sports-specific training is more effective than general training in improving sport-specific performance [12]. Specifically, our court-based SIT protocol involved movements that were more similar to those performed during a tennis match, including multidirectional running and changes of direction. These movements require a higher degree of neuromuscular control (e.g., type of muscle contraction, the demand on acceleration and deceleration, and lateral movements) and coordination, which are critical components of tennis-specific endurance. Furthermore, both SIT and ET develop the aerobic energy supply system but SIT also develops the anaerobic energy supply system and enhances the fatigue resistance of the central nervous system [17]. In this regard, a recent study conducted by Kilit and Arslan [36] showed that both HIIT and on-court tennis drill training could enhance cardiorespiratory endurance significantly ($\dot{\text{V}}$O_2max_ increased between 5.2% and 5.5% in both training conditions while HIIT exhibited a much better running time in 400-m test) whereas the on-court tennis training group demonstrated significantly better agility and technical performance than the HIIT group. Similarly, Fernandez-Fernandez et al. [37] found that the mixed program combining both the HIIT and tennis drills could potentially yield a better performance gain than using sport-specific training drills alone. Given the intense HIIT nature of court-based SIT and its high specificity to tennis movements and biomechanical demands, our court-based SIT was shown to potentially share the beneficial effects and characteristics of both HIIT and on-court tennis training proposed in previous studies.

Despite the removal of the need for stroke quality and most of the energy demand from the upper body in hitting the real tennis ball during the HIT&TURN test, it highly mimics the intermittent nature and both the neuromuscular and biomechanical demands of tennis [26]. Although a traditional lab-based $\dot{\text{V}}$O_2max_ test is regarded as a golden standard to verify the aerobic performance of individuals, a previous study showed only a moderate correlation between $\dot{\text{V}}$O_2max_ and the level of play (i.e., ITN level) in competitive male tennis players [38]. The laboratory-based test conducted under strictly controlled conditions, therefore, may not provide a complete representation of real-world performance [39]. On the other hand, the mechanical efficiency (i.e., also known as “exercise efficiency’) of an athlete should be concurrently considered in the competitive sports context [40]. As the HIT&TURN test may serve to reflect aerobic efficiency and actual tennis endurance, players with the same aerobic capacity (i.e., $\dot{\text{V}}$O_2max_) but a higher HIT&TURN score are considered to be more “efficient” than those who only performed well in lab-based performance. Taken together, the improvements in tennis-specific endurance observed in this study are likely attributed to the sports-specific nature of our SIT program, providing further insight into the optimal training regimen for tennis players. Future studies may focus on the comparison between equipment-based and court-based SIT on the improvements in HIT&TURN as well as the neuromuscular performance variables of tennis players to further understand the actual source and distribution of adaptions taking place in these SIT protocols.

This study demonstrates notable strengths, including the recruitment of high-level tennis players and the utilization of both laboratory and tennis-specific field tests. These factors significantly enhance the external validity of the findings. However, it is important to acknowledge several limitations. Firstly, the present study design did not investigate the moderation effects of specific variables within the SIT regimen, such as sprint length and the number of sprints performed per session. This decision was made to maintain a focus on targeting high-end capacities (all-out efforts) during the session while optimizing the overall time efficiency of the training tool. Given that the duration of the six-week intervention resembles the typical length of a training block (i.e., mesocycle) involved in the periodization of competitive tennis players, our study provides the clear training effects of the court-based SIT and practical implications for program planning by the conditioning coaches. To further capture the full potential impacts on the measured outcomes in different training blocks of a season or macrocycle, future studies may consider extending the intervention across the entire macrocycle or comparing the effectiveness of the two regimens when implemented in different periodization phases. Furthermore, our study did not explore the impact of dietary and other lifestyle factors on the SIT intervention. Future studies can focus on the investigation of these variables to provide a more comprehensive understanding of the optimum parameters of SIT on the aerobic capacity and tennis-specific endurance for competitive tennis players. Apart from aerobic performance variables, assessments in neuromuscular performance such as strength, power, and balance for both SIT and ET, could also be included to further evaluate the effectiveness of these two training regimens.

## CONCLUSIONS

Compared with traditional ET, court-based SIT can be a time-efficient strategy to improve aerobic capacity and tennis-specific endurance without requiring specialized equipment for competitive tennis players. Our findings support the coaches and athletes in adopting court-based SIT to improve both the physical fitness levels and enhance the on-court performance of competitive tennis players. Further studies are needed to investigate the long-term effects of SIT on tennis-specific performance and to determine the optimal SIT protocol for competitive tennis players.
